# Blood plasma trimethylamine N-oxide and related metabolites and asthenozoospermia odds: a hospital-based matched case–control study in China

**DOI:** 10.1093/hropen/hoaf045

**Published:** 2025-08-18

**Authors:** Ze Xing, Meng-Meng Xie, Hui-Han Wang, Qi Cui, Xiao-Bin Wang

**Affiliations:** Center of Reproductive Medicine, Shengjing Hospital of China Medical University, Shenyang, P.R. China; Department of Obstetrics and Gynecology, Shengjing Hospital of China Medical University, Shenyang, P.R. China; Department of Obstetrics and Gynecology, Shengjing Hospital of China Medical University, Shenyang, P.R. China; Department of Hematology, Shengjing Hospital of China Medical University, Shenyang, P.R. China; Department of Laboratory Medicine, Shengjing Hospital of China Medical University, Shenyang, P.R. China; Center of Reproductive Medicine, Shengjing Hospital of China Medical University, Shenyang, P.R. China; Department of Obstetrics and Gynecology, Shengjing Hospital of China Medical University, Shenyang, P.R. China

**Keywords:** asthenozoospermia, case–control study, China, sperm quality, trimethylamine N-oxide

## Abstract

**STUDY QUESTION:**

Are blood plasma trimethylamine N-oxide (TMAO) and related metabolites linked to the odds of asthenozoospermia?

**SUMMARY ANSWER:**

Increased blood plasma TMAO levels were positively associated with the odds of asthenozoospermia, while elevated levels of choline and L-carnitine were related to reduced asthenozoospermia odds, implying that TMAO and its related metabolites might play an important role in the development of asthenozoospermia.

**WHAT IS KNOWN ALREADY:**

Sperm motility and concentration are profoundly impaired by excessive reactive oxygen species (ROS). A positive correlation has been established between ROS levels and TMAO, which is regarded as a key regulatory factor for initiating mitochondrial ROS production. However, the precise interplay between TMAO and its metabolites and sperm quality remains inconclusive and insufficient.

**STUDY DESIGN, SIZE, DURATION:**

This case–control study was conducted from June 2020 to December 2020. A total of 314 pairs of asthenozoospermia cases and normozoospermia controls, matched based on age, BMI, and smoking status, were included.

**PARTICIPANTS/MATERIALS, SETTING, METHODS:**

Blood plasma levels of TMAO and five related metabolites, such as choline, betaine, L-carnitine, methionine, and dimethylglycine, were measured using a liquid chromatography system coupled with tandem mass spectrometry. Multivariable conditional logistic regression models were used to estimate the odds ratios (ORs) and corresponding 95% CIs.

**MAIN RESULTS AND THE ROLE OF CHANCE:**

Compared with the lowest quartile, a significant association was observed between blood plasma TMAO level (OR = 1.80, 95% CI = 1.16–2.81) and the odds of asthenozoospermia for the highest quartile. In contrast, choline (OR = 0.59, 95% CI = 0.37–0.92) and L-carnitine (OR = 0.58, 95% CI = 0.37–0.90) levels were significant inversely associated with the odds of asthenozoospermia. Additionally, for each per SD change, significant dose–response relationships were noted with increased odds of asthenozoospermia linked to elevated TMAO (OR = 1.31, 95% CI = 1.12–1.55), as well as L-carnitine (OR = 0.79, 95% CI = 0.67–0.93) and total methyl donors exposure (OR = 0.82, 95% CI = 0.70–0.96) levels.

**LIMITATIONS, REASONS FOR CAUTION:**

We cannot infer causality from this study due to the case–control study. Since the current study was conducted on a population of Chinese men, the extrapolated results may not accurately reflect other regions or populations. As blood plasma TMAO and its metabolites were measured at a single time point and may not accurately represent long-term concentrations, the enduring effects on sperm quality may not be fully captured. Another limitation of the current study lies in its relatively modest sample size, which may have been insufficient to reach statistical power in subgroup analyses.

**WIDER IMPLICATIONS OF THE FINDINGS:**

This study indicated that elevated blood plasma TMAO levels were associated with increased odds of asthenozoospermia, while higher concentrations of choline and L-carnitine decreased asthenozoospermia odds. Our results provide novel evidence that TMAO and its metabolites may serve as potential biomarkers for asthenozoospermia.

**STUDY FUNDING/COMPETING INTEREST(S):**

No funding was received for this study. All authors have no conflict of interest to declare.

**TRIAL REGISTRATION NUMBER:**

N/A.

WHAT DOES THIS MEAN FOR PATIENTS? Male infertility is a global public health issue, and reduced sperm motility, medically referred to as ‘asthenozoospermia’, is one of the main causes of male infertility, accounting for 20–40% of male infertility cases. The debate on why semen quality is diminishing has persisted for decades, with several physiological, environmental, and genetic factors all being considered. Oxidative stress, which can be thought of as an imbalance between damaging types of oxygen, referred to by scientists as reactive oxygen species, and a body’s ability to counteract the damage they inflict, is one of the primary mechanisms of male infertility.

Excessive reactive oxygen species can severely impair sperm motility and concentration, and a positive correlation was previously established between their levels and a substance investigated in this study, known as trimethylamine N-oxide (TMAO). TMAO is produced through the metabolism of substances such as choline, phosphatidylcholine, L-carnitine, and betaine in the diet by gut microbes. Subsequently, it is absorbed into the bloodstream and can be detected in blood plasma. However, regarding the odds of asthenozoospermia, the role of TMAO and its metabolites (related substances), including choline, betaine, L-carnitine, methionine, dimethylglycine, and total methyl donors (the sum of choline, betaine, and methionine), remains inconclusive and insufficient.

Hence, we performed a hospital-based matched case–control study involving 314 pairs of patients (cases) with asthenozoospermia matched with healthy study participants (controls) with normal sperm to make a thorough inquiry into this topic.

Our results showed that increased TMAO levels in blood were correlated with increased odds of asthenozoospermia, whereas increased choline and L-carnitine levels were linked to lower odds of asthenozoospermia. Further analyses suggested that eating more red meat may enhance the effects of TMAO-related substances on asthenozoospermia.

Therefore, for patients with asthenozoospermia, reducing the consumption of red meat may help mitigate the negative impacts associated with the combined action of red meat and these metabolites on sperm motility. If further studies, including those in non-Chinese populations, confirm our findings, there may be potential to improve sperm quality by focusing on blood plasma TMAO and its associated metabolites as key indicators.

## Introduction

The global disease burden of infertility has continued to increase, progressively emerging as a significant health challenge (Sun *et al.*, 2019b). Recognition of the critical significance of male reproductive function has increased in recent years (Levine *et al.*, 2018). The causes of infertility include low sperm counts, poor sperm quality, endocrinopathies, lifestyle factors (such as smoking and obesity), congenital anatomical factors, gonadotoxic exposures, and aging (Eisenberg *et al.*, 2023), with asthenozoospermia being one of the most common causes (Chemes, 2000; Huynh *et al.*, 2002; Curi *et al.*, 2003). A study conducted in Argentina revealed that asthenozoospermia was a prevalent cause of male infertility, with a prevalence of 18.71%. Furthermore, the combined prevalence of asthenozoospermia with oligospermia and/or teratozoospermia rose to 63.13% (Curi *et al.*, 2003). An analysis of 38 905 infertile male patients in China found that 50.5% were diagnosed with asthenozoospermia, with the prevalence steadily rising from 2008 to 2016 (Wu *et al.*, 2021). Research on the causes of asthenozoospermia and strategies to prevent further disruption of male reproductive health are urgently needed.

Choline, an essential nutrient, plays a crucial role in the proper functioning of the liver, muscles, and brain, as well as in lipid metabolism and the composition and repair of cell membranes (Zeisel and da Costa, 2009; Wallace, 2018). Gut microbiota is recognized as the second genome of the human body (Jiao *et al.*, 2022), with previous studies highlighting its contribution to human metabolism and health (Koeth *et al.*, 2013; Tang *et al.*, 2013, 2019). The gut microbiota converts dietary precursors such as choline, phosphatidylcholine, L-carnitine, and betaine into trimethylamine (TMA), which is subsequently metabolized by the hepatic enzyme flavin monooxygenase-3 to produce TMAO (Li *et al.*, 2017; Wang *et al.*, 2023). Previous research indicates that diet is a key factor in regulating TMAO and its related metabolites. The Mediterranean diet, rich in cereals, fruits, vegetables, and legumes, has been linked to lower TMAO levels (De Filippis *et al.*, 2016; Castañón-Apilánez *et al.*, 2024; Latif *et al.*, 2025; Theodoridis *et al.*, 2025). Conversely, red meats like beef and pork are major dietary sources of L-carnitine and choline. Gut microbiota converts L-carnitine and choline into TMA, which is then oxidized in the liver and released as TMAO, thereby increasing blood plasma TMAO concentrations (Koeth *et al.*, 2013; Guerreiro *et al.*, 2025). Another diet, the very low-calorie ketogenic diet (VLCKD), which is known for its role in weight loss and the generation of anti-inflammatory ketone bodies, has drawn attention for its therapeutic effect in male sexual dysfunction (Condorelli *et al.*, 2022). It has been reported that the levels of TMAO significantly decrease after intervention with the VLCKD (Verde *et al.*, 2024a, 2024b; Theodoridis *et al.*, 2025). Moreover, a plant-based diet and a high-dairy diet have been linked to a reduction in TMAO concentration, while a high-protein and high-fat diet is associated with an increase in TMAO concentration (Theodoridis *et al.*, 2025). Notably, restricting total energy consumption has been shown to effectively reduce both TMAO and choline levels (Heianza *et al.*, 2019; Battillo and Malin, 2023). Lifestyle interventions, such as a hypocaloric diet and regular physical activity, appear to be effective in reducing blood plasma TMAO (Erickson *et al.*, 2019; Theodoridis *et al.*, 2025). However, the effect of lifestyle interventions on TMAO is controversial. There are also studies indicating that dietary modifications and following exercise recommendations show no significant impact on TMAO levels (Randrianarisoa *et al.*, 2016; Troseid *et al.*, 2016). Previous studies have demonstrated that TMAO additionally stimulates reactive oxygen species (ROS) generation, particularly mitochondrial ROS (Chen *et al.*, 2017), indicating a positive correlation between ROS levels and TMAO (Darbandi *et al.*, 2019). Excessive ROS can significantly impair total sperm motility and sperm concentration (Darbandi *et al.*, 2019). Additionally, Mateo-Otero *et al.* (2021) identified an inverse relationship between TMAO concentrations in pig seminal plasma and viable sperm with high membrane destabilization, suggesting TMAO could serve as a potential biomarker of sperm function. Increasing evidence links oxidative stress (OS) to the pathogenesis of male infertility (Bisht *et al.*, 2017; Agarwal *et al.*, 2021a, 2021b), and the term ‘Male Oxidative Stress Infertility’ has been coined to describe these male-specific effects (Agarwal *et al.*, 2019). Choline, betaine, L-carnitine, methionine, and dimethylglycine (DMG) all contribute to combating OS (Zhao *et al.*, 2018; Bai *et al.*, 2019; Khedr and Werida, 2022; Dong *et al.*, 2024), but their potential connection to asthenozoospermia remains unexplored.

In this study, we aimed to investigate the association between blood plasma TMAO and related metabolites, such as choline, betaine, L-carnitine, methionine, DMG, and total methyl donors (the sum of choline, betaine, and methionine), and asthenozoospermia odds in a case–control study.

## Materials and methods

### Study design and subjects

**Figure 1.**
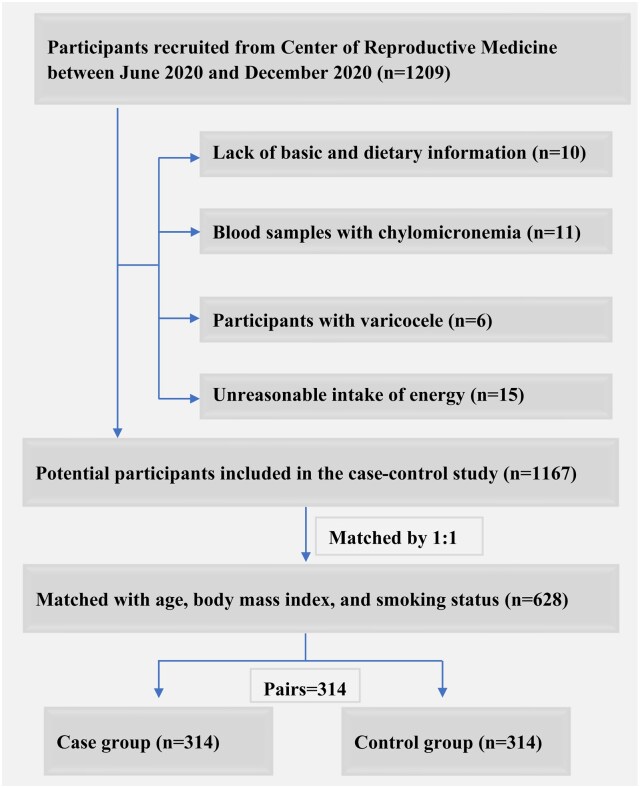
Flow diagram of the selection of men for the hospital-based matched case–control study to assess the relations of blood plasma trimethylamine N-oxide and related metabolites and asthenozoospermia odds.

This case–control study was conducted from June 2020 to December 2020. Male patients attending the Center of Reproductive Medicine at Shengjing Hospital of China Medical University were included in the study. In total, 597 asthenozoospermia cases and 612 healthy controls were initially recruited, prior to evaluating all exclusion criteria and case–control matching (Fig. 1). All participants underwent sperm analysis and physical examinations and completed self-administered questionnaires. Trained nurses reviewed all the completed questionnaires and promptly contacted participants to correct any missing or unclear responses. All participants provided written informed consent upon enrollment in the study. This study was approved by the ethics committee of Shengjing Hospital of China Medical University and was conducted in accordance with the principles of the Declaration of Helsinki (Goodyear *et al.*, 2007; World Medical Association, 2013) as well as the Strengthening the Reporting of Observational Studies in Epidemiology statement (von Elm *et al.*, 2007).

We excluded participants who failed to provide complete basic and dietary information (e.g. age, height, weight, household income, physical activity level, smoking and drinking alcohol status, occupational status, education level, total energy intake, total red meat intake, and sexual abstinence time) (n = 10). Participants who provided blood samples with chylomicronemia were excluded (n = 11). Those with a history of varicocele, a known confounding factor, were excluded as well (n = 6) (Eslamian *et al.*, 2012; Jensen *et al.*, 2017; Liu *et al.*, 2021; Lv *et al.*, 2022). Additionally, individuals with implausible total energy intake values (>6000 or <800 kcal/day) were removed from the analysis (n = 15) (Shi *et al.*, 2018). Each asthenozoospermia case was 1:1 matched with a healthy control based on age, BMI, and smoking status. In the end, a total of 314 cases and 314 matched controls were included in the final analysis (Fig. 1). This sample size of 628 men met the required number to achieve adequate statistical power, as determined through Power Analysis & Sample Size (PASS, NCSS, LLC; Kaysville, UT, USA) 11.0 software.

### Semen collection and analysis

The methods for semen collection and analysis have been thoroughly described elsewhere (Liu *et al.*, 2021, 2022; Wang *et al.*, 2021a). All participants were instructed to abstain from ejaculation for 3–7 days prior to providing a semen sample. The samples were collected via masturbation into a sterile container, ensuring no contact with condoms or lubricants. After collection, the semen was allowed to liquefy for no more than 60 min before analysis. Sperm parameters were assessed using a WLJY9000 analysis instrument (Beijing Weili New Century Science & Tech. Dev. Co. Ltd, Beijing, China). Each semen sample was examined twice by two experienced technicians, and the reference values of normal sperm were identified according to the WHO criteria (World Health Organization, 2021).

### Definition of asthenozoospermia

Asthenozoospermia was diagnosed based on the criteria outlined in the fifth edition of the World Health Organization laboratory manual for the examination and processing of human semen (World Health Organization, 2010). It was defined as having a total motility (progressive+non-progressive) of less than 40%, which includes rapidly progressive, slowly progressive, and non-progressive sperm, as well as progressive motility of less than 32%, encompassing both rapidly and slowly progressive motility, within 60 min of ejaculation over the past 3 months. The total sperm count and the percentage of morphologically normal spermatozoa were above the reference values for the lower limits. The control group consisted of men with normal sperm parameters, including ≥39 million sperm per ejaculate, ≥15 million sperm per milliliter, ≥40% total motility, ≥32% progressive motility, and ≥4% normal morphology.

### Data collection

Demographic, socio-economic features, smoking status, alcohol intake, physical activity levels, lifestyle factors, dietary data, and sexual abstinence time were obtained at baseline through self-administered questionnaires. Anthropometric data were collected by trained investigators using standardized techniques and equipment. Moreover, clinical data were extracted from digital medical records, and blood plasma samples were collected from each participant at the time of recruitment. Body weight and height were measured during physical examinations. BMI was calculated by dividing weight (kg) by height squared (m^2^). Physical activity was expressed as metabolic equivalent (MET) hours per week, determined by multiplying the frequency of physical activity by its corresponding MET coefficient (Du *et al.*, 2013).

In addition, dietary intake information was collected using a 110-item food frequency questionnaire (Cui* et al.*, 2023; Huang *et al.*, 2023; Zhao *et al.*, 2023), and our previous studies have demonstrated its reliability and validity (Liu *et al.*, 2021; Wang *et al.*, 2021b; Cui *et al.*, 2022). All patients reported how often they consumed each food in the year prior to their diagnosis of asthenozoospermia. Intake frequency was divided into seven categories: ‘almost never’, ‘2–3 times a month’, ‘once a week’, ‘2–3 times a week’, ‘4–6 times a week’, ‘once a day’, ‘≥2 times a day’. For most food groups, the reproducibility coefficients (Spearman and intraclass correlation coefficients) were above 0.5, and the Spearman correlation coefficients ranged between 0.3 and 0.7 for most food groups between the questionnaire and weighed diet records. Based on the China Food Composition Table, the frequency of consumption of each food was multiplied by the nutrient content of the designated portions to determine the daily intake of each food (Hu *et al.*, 2018; Yang *et al.*, 2018).

### TMAO and its related metabolites measurement

At baseline, a venous blood sample was collected from each subject and centrifuged at 1800 *g* for 10 min. Blood plasma was separated and stored at −80°C for analysis. TMAO and its related metabolites were extracted from blood plasma by solid phase extraction and measured by a liquid chromatography system coupled with tandem mass spectrometry (LC-MS/MS) (Wang *et al.*, 2014; Kalagi *et al.*, 2022; Kijpaisalratana *et al.*, 2023). Blood plasma samples were analyzed in batches, and samples from cases and controls were mixed and anonymized prior to delivery to the laboratory, ensuring a fully blinded assessment while accounting for any potential batch differences in the analysis process. As an additional quality control measure to ensure the accuracy, consistency, and repeatability of the method, all laboratory procedures were completed in 2024, and ∼10% of the blood plasma samples were subjected to repeat analysis. The coefficient of variation in the laboratory for the blood plasma TMAO measurements was consistently below 10%.

### Statistical analyses

Categorical variables were expressed as numbers with percentages. Mean with SD was utilized for normally distributed continuous variables, and median with quartile range was used for non-normally distributed continuous variables. Student’s *t*-tests (normally distributed) or the Wilcoxon signed rank test (non-normally distributed) were used for continuous variables, and the chi-square test was used for categorical variables to compare the differences between the case and the control group. Spearman correlation analysis was employed to assess the associations between blood plasma levels of TMAO and its metabolites. Blood plasma TMAO and its metabolite levels were categorized into quartiles according to their distribution among the controls. Total methyl donors were the sum of choline, betaine, and methionine (Huang *et al.*, 2020). A multivariable conditional logistic regression model was applied to estimate the odds ratios (ORs) and 95% CIs with the lowest quartile as the reference group. The selection of confounders was based on previous literature and guided by the directed acyclic graph (Supplementary Fig. S1) (Tennant *et al.*, 2021). Accordingly, adjusted potential confounders included educational level (junior secondary or below, senior high school/technical secondary school, or junior college/university or above), total energy intake (kcal/d), alcohol intake (yes or no), sexual abstinence time (days), serum creatinine (µmol/l), and total red meat intake (g/day), with Model 1 adjusted for educational level, total energy, and alcohol intake and Model 2 further adjusted for serum creatinine, total red meat intake, and sexual abstinence time based on Model 1. The linear trend tests were examined by using the median value of each quartile. We also modeled each variable as a continuous variable and derived ORs and 95% CIs.

Subgroup analyses were carried out based on alcohol intake (yes and no), total energy intake (below median and above median), and total red meat intake (below median and above median). The potential interactions between TMAO and related metabolites and these stratified variables were analyzed by using cross-product terms in the multivariable models.

Sensitivity analyses were conducted to assess the robustness of the results. Drawing on clinical significance and published literature, two additional models were conducted based on the Model 2: (i) further adjusted for physical activity (Pingitore *et al.*, 2015; Sun *et al.*, 2019a); and (ii) further adjusted for occupation (Daoud *et al.*, 2017; Wu *et al.*, 2021). All analyses were performed by SAS 9.4 (SAS Institute, Cary, NC, USA). A two-sided alpha level of 0.05 was chosen for the statistical significance of all the analyses.

## Results

### Baseline characteristics of the population

Table 1 summarizes the general characteristics of the 314 pairs of subjects. When compared with the controls, participants with asthenozoospermia exhibited significantly lower sperm concentrations and total sperm counts, and lower percentages with progressive motility, total motility, and normal sperm morphology (all *P *< 0.05).

**Table 1.** Characteristics of the participants.

Characteristics	Control group (n = 314)	Case group (n = 314)	*P-*value
**Age (years)**	32 (30–35)	32 (30–36)	0.57
**BMI (kg/m²)**	25.82 (23.36–28.73)	26.12 (23.94–28.40)	0.43
**Smoking status**			1.00
No	163 (51.91)	163 (51.91)	
Yes	151 (48.09)	151 (48.09)	
**Alcohol drinking**			0.25
No	182 (57.96)	197 (62.74)	
Yes	132 (42.04)	117 (37.26)	
**Occupation**			0.81
Employed	173 (55.10)	177 (56.37)	
Unemployed	141 (44.90)	137 (43.63)	
**Educational level**			0.48
Junior secondary or below	75 (23.89)	65 (20.70)	
Senior high/technical secondary	37 (11.78)	45 (14.33)	
Junior college/university or above	202 (64.33)	204 (64.97)	
**Serum creatinine (µmol/l)**	77.10 (62.51–93.32)	76.67 (64.06–93.06)	0.95
Physical activity (MET*h/d)	125.53 (95.55–223.87)	131.33 (101.33–213.27)	0.29
**Total energy intake (kcal/d)**	1685.05 (1402.45–2117.06)	1756.55 (1412.89–2196.67)	0.24
**Total red meat intake (g/day)**	82.87 (59.99–96.99)	79.85 (49.24–95.37)	0.05
**Sexual abstinence time (days)**	4 (3–5)	4 (3–5)	1.00
**Semen parameters**			
Ejaculate volume (ml)	3.20 (2.50–4.20)	3.50 (2.80–4.40)	0.23
Sperm concentration (10^6^/ml)	63.99 (42.87–85.23)	48.93 (31.73–71.15)	<0.001
Total sperm count (10^6^/ml)	214.94 (139.38–291.06)	166.26 (106.64–249.85)	<0.001
Progressive motility (%)	43.03 (38.43–49.79)	22.37 (15.27–28.21)	<0.001
Total motility (%)	53.61 (46.89–62.20)	27.79 (19.82–35.12)	<0.001
Normal sperm morphology (%)	6.00 (4.00–8.00)	5.00 (4.00–7.00)	<0.001

Values are means (SDs) for normally distributed continuous variables (age at diagnosis), medians (interquartile ranges) for skewed distributed continuous variables (BMI, physical activity, serum creatine, total energy intake, and total red meat intake), and values are numbers (percentages) for categorical variables. *P-*values <0.05 were considered statistically significant.

*P-*values were determined with Student’s *t*-tests (normally distributed) or Wilcoxon signed rank test (non-normally distributed) for continuous variables, and chi-square test for categorical variables to compare the differences between the case and the control group. All statistical tests are two sided.

MET, metabolic equivalent.

### Concentrations of blood plasma TMAO and related metabolites

**Figure 2.**
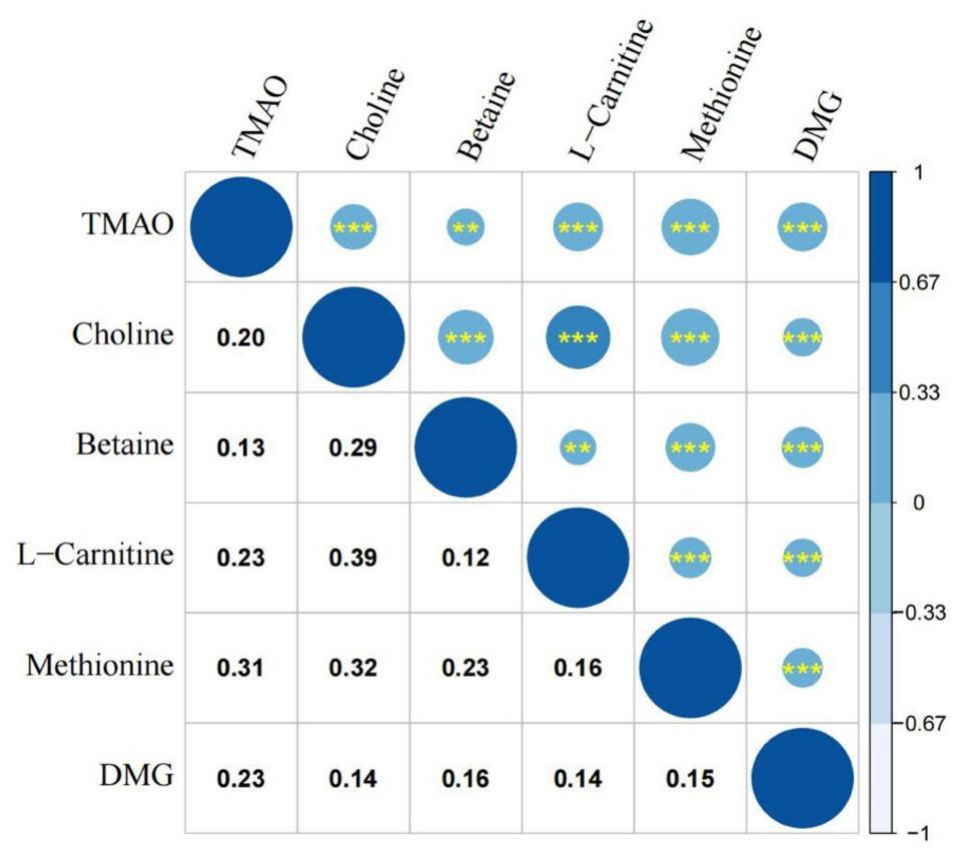
**Spearman correlations among blood plasma trimethylamine N-oxide and related metabolites.** The correlation among blood plasma TMAO and related metabolites is statistically significant (**P < 0.01; ***P < 0.001). The numbers below the navy-blue circles in the figure represent the magnitude of the Spearman’s rank correlation coefficients. The scale on the right side reflects the strength and direction of the linear relationship between variables. The closer the absolute value of the coefficient is to 1, the stronger the linear relationship is; a positive coefficient indicates a positive correlation; a negative coefficient indicates a negative correlation; a coefficient of 0 indicates the absence of a linear relationship. DMG, dimethylglycine; TMAO, trimethylamine N-oxide.

The distributions of blood plasma TMAO and related metabolites concentrations are shown in Table 2. The blood plasma TMAO concentration was elevated in the case group compared with the control group (*P *< 0.05). Conversely, the concentrations of choline, L-carnitine, methionine, and methyl donors in blood plasma were relatively higher in the control group than in the case group (all *P *< 0.05). Spearman correlation analysis identified associations between the concentrations of TMAO and its related metabolites (Fig. 2). Notably, the strongest correlation was observed between blood plasma choline and L-carnitine levels (Spearman’s rank correlation coefficients = 0.39).

**Table 2.** Blood plasma TMAO and related metabolites in the study population.

	CV (%)	Median (IQR) (µmol/l)			*P*-value
		Total population	Case group	Control group	
**TMAO**	8.4	7.95 (4.50–11.50)	8.49 (7.51–4.92)	7.10 (6.62–4.18)	<0.01
**Choline**	5.7	14.68 (11.10–19.36)	14.15 (8.55–10.66)	15.09 (8.24–11.71)	0.01
**Betaine**	6.9	39.12 (30.31–48.91)	37.81 (20.12–28.89)	39.91 (17.21–31.34)	0.31
**L-carnitine**	4.3	43.75 (33.40–57.03)	42.22 (24.69–30.73)	45.97 (22.05–36.03)	<0.01
**Methionine**	8.8	28.56 (20.70–36.65)	26.65 (16.06–19.78)	29.63 (15.60–21.42)	0.01
**DMG**	7.1	9.19 (5.97–12.51)	8.69 (6.23–5.90)	9.35 (6.54–6.08)	0.29
**Methyl donors**	**–**	82.54 (67.89–97.45)	79.40 (28.40–67.30)	85.18 (29.80–69.79)	0.01

CV, coefficient of variation; DMG, dimethylglycine; IQR, interquartile range; TMAO, trimethylamine-N-oxide.

*P-*values < 0.05 were considered statistically significant.

*P*-values were obtained from the Wilcoxon rank sum test between the case group and the control group.

### Associations of blood plasma TMAO and its related metabolites with the odds of asthenozoospermia

Table 3 shows the associations between blood plasma TMAO and related metabolites and the odds of asthenozoospermia. In multivariate-adjusted models, TMAO levels at the highest quartile were significantly associated with increased odds of developing asthenozoospermia compared with the lowest quartile (OR = 1.80, 95% CI = 1.16–2.81). In contrast, blood plasma concentrations of choline (OR = 0.59, 95% CI = 0.37–0.92) and L-carnitine (OR = 0.58, 95% CI = 0.37–0.90) were significantly inversely associated with the odds of asthenozoospermia, when comparing the highest quartile with the lowest quartile. Furthermore, significant dose–response relationships were observed, with increased odds of asthenozoospermia linked to higher TMAO levels (OR = 1.31, 95% CI = 1.12–1.55) for each per SD change. Additionally, decreased odds of asthenozoospermia were associated with lower levels of L-carnitine (OR = 0.79, 95% CI = 0.67–0.93) and total methyl donors exposure (OR = 0.82, 95% CI = 0.70–0.96).

**Table 3.** Odds ratios and 95% CIs for the association between blood plasma TMAO and related metabolites with asthenozoospermia.

	Quartile 1	Quartile 2	Quartile 3	Quartile 4	*P* for trend	**Per SD increment** ^a^
**TMAO (µmol/l)**	≤ 4.18	4.18–7.10	7.10–10.80	>10.80		
Cases/controls	66/79	58/78	77/79	113/78		
Crude model	1.00 (Ref)	0.89 (0.56–1.43)	1.17 (0.74–1.84)	1.73 (1.12–2.68)	<0.05	1.29 (1.10–1.52)
Model 1	1.00 (Ref)	0.88 (0.55–1.41)	1.15 (0.73–1.81)	1.75 (1.13–2.71)	<0.05	1.30 (1.11–1.53)
Model 2	1.00 (Ref)	0.88 (0.54–1.42)	1.18 (0.74–1.86)	1.80 (1.16–2.81)	<0.05	1.31 (1.12–1.55)
**Choline (µmol/l)**	≤ 11.71	11.71–15.09	15.09–19.95	>19.95		
Cases/controls	101/79	68/78	86/79	59/78		
Crude model	1.00 (Ref)	0.68 (0.44–1.06)	0.85 (0.56–1.30)	0.59 (0.38–0.93)	0.06	0.82 (0.70–0.96)
Model 1		0.67 (0.43–1.05)	0.84 (0.55–1.29)	0.58 (0.37–0.92)	0.06	0.82 (0.70–0.96)
Model 2	1.00 (Ref)	0.68 (0.44–1.06)	0.82 (0.53–1.26)	0.59 (0.37–0.92)	0.06	0.82 (0.70–0.96)
**Betaine (µmol/l)**	≤ 31.34	31.34–39.91	39.91–48.55	>48.55		
Cases/controls	101/79	69/78	61/79	83/78		
Crude model	1.00 (Ref)	0.69 (0.45–1.07)	0.60 (0.39–0.94)	0.83 (0.54–1.28)	0.32	0.93 (0.79–1.08)
Model 1	1.00 (Ref)	0.69 (0.44–1.07)	0.61 (0.39–0.95)	0.83 (0.54–1.27)	0.31	0.92 (0.79–1.08)
Model 2	1.00 (Ref)	0.68 (0.44–1.05)	0.60 (0.38–0.95)	0.83 (0.54–1.29)	0.33	0.92 (0.78–1.08)
**L-carnitine (µmol/l)**	≤ 36.03	36.03–45.97	45.97–58.08	>58.08		
Cases/controls	118/79	66/78	65/79	65/78		
Crude model	1.00 (Ref)	0.57 (0.37–0.88)	0.55 (0.36–0.85)	0.56 (0.36–0.86)	<0.05	0.79 (0.67––0.92)
Model 1	1.00 (Ref)	0.56 (0.36–0.87)	0.56 (0.36–0.86)	0.57 (0.37–0.89)	<0.05	0.79 (0.68–0.93)
Model 2	1.00 (Ref)	0.56 (0.36–0.87)	0.55 (0.35–0.85)	0.58 (0.37–0.90)	<0.05	0.79 (0.67–0.93)
**Methionine (µmol/l)**	≤ 21.42	21.42–29.63	29.63–37.02	>37.02		
Cases/controls	95/79	86/78	62/79	71/78		
Crude model	1.00 (Ref)	0.92 (0.60–1.41)	0.65 (0.42–1.02)	0.76 (0.49–1.17)	0.11	0.82 (0.70–0.96)
Model 1	1.00 (Ref)	0.92 (0.60–1.41)	0.65 (0.42–1.02)	0.76 (0.49–1.19)	0.12	0.82 (0.70–0.97)
Model 2	1.00 (Ref)	0.92 (0.59–1.41)	0.66 (0.42–1.04)	0.77 (0.49–1.20)	0.13	0.83 (0.70–0.97)
**DMG (µmol/l)**	≤ 6.08	6.08–9.35	9.35–12.62	>12.62		
Cases/controls	85/79	80/78	80/79	69/78		
Crude model	1.00 (Ref)	0.95 (0.62–1.48)	0.94 (0.61–1.46)	0.82 (0.53–1.28)	0.62	0.92 (0.78–1.07)
Model 1	1.00 (Ref)	0.96 (0.62–1.49)	0.92 (0.60–1.43)	0.82 (0.53–1.29)	0.62	0.92 (0.78–1.07)
Model 2	1.00 (Ref)	0.93 (0.60–1.46)	0.93 (0.60–1.45)	0.84 (0.53–1.31)	0.60	0.92 (0.78–1.08)
**Methyl donors (µmol/l)**	≤ 69.79	69.79–85.18	85.18–99.59	>99.59		
Cases/controls	95/79	91/78	62/79	66/78		
Crude model	1.00 (Ref)	0.97 (0.63–1.48)	0.65 (0.42–1.02)	0.70 (0.45–1.10)	<0.05	0.82 (0.70–0.96)
Model 1	1.00 (Ref)	0.95 (0.62–1.46)	0.63 (0.40–0.99)	0.71 (0.45–1.10)	<0.05	0.82 (0.70–0.96)
Model 2	1.00 (Ref)	0.92 (0.59–1.41)	0.62 (0.39–0.97)	0.70 (0.45–1.09)	<0.05	0.82 (0.70–0.96)

Quartile values for each model represent OR (95% CI). *P-*values < 0.05 were considered statistically significant.

DMG, dimethylglycine; OR, odds ratios; Ref, reference; TMAO, trimethylamine-N-oxide.

Model 1: Adjusted for educational level, total energy, and alcohol intake.

Model 2: Further adjusted for serum creatinine, total red meat intake, and sexual abstinence time based on Model 1.

a The SD of TMAO, choline, betaine, L-carnitine, methionine, DMG, and methyl donors were 4.23 µmol/l, 4.91 µmol/l, 11.69 µmol/l, 14.13 µmol/l, 10.69 µmol/l, 4.23 µmol/l, and 20.10 µmol/l, respectively.

### Subgroup analysis, interaction, and sensitivity analysis

**Figure 3.**
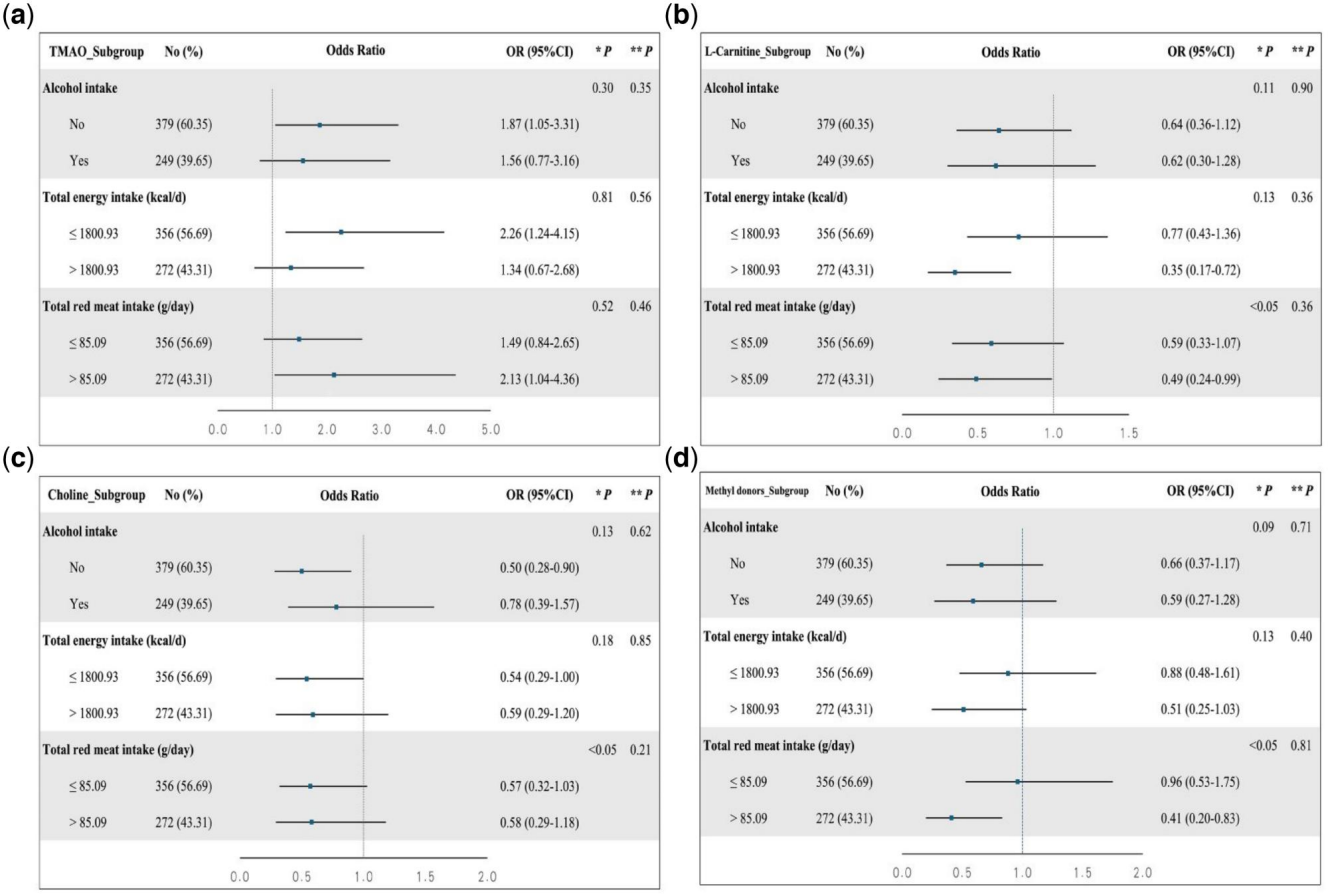
**Subgroup analyses of the associations between TMAO, L-carnitine, choline, and methyl donors and the odds of asthenozoospermia.** (**A**) Subgroup analyses of the association between TMAO and the odds of asthenozoospermia. (**B**) Subgroup analyses of the association between L-carnitine and the odds of asthenozoospermia. (**C**) Subgroup analyses of the association between choline and the odds of asthenozoospermia. (**D**) Subgroup analyses of the association between methyl donors and the odds of asthenozoospermia. kcal/d, total energy intake of 1 day; OR, odds ratio; TMAO, trimethylamine N-oxide. * *P* for multiplicative interaction. ** *P* for additive interaction. *P-*values < 0.05 were considered statistically significant.

To further analyze the relationship between blood plasma TMAO and related metabolites and the odds of asthenozoospermia, subgroup analyses were performed based on demographic and clinical characteristics (Supplementary Table S1). In terms of the odds of asthenozoospermia, multiplicative interactions were observed between total red meat intake and choline, betaine, L-carnitine, methionine, DMG, as well as total methyl donors (all *P *< 0.05) (Supplementary Table S1). Regarding the relationship between TMAO and L-carnitine levels and the odds of asthenozoospermia, most subgroup analyses were oriented in the same direction as the main findings (Fig. 3). Among the other metabolites evaluated, choline and total methyl donors levels showed statistical significance across several specific subgroups (Fig. 3), while no subgroup analysis of betaine, methionine, and DMG exhibited statistically significant outcomes (Supplementary Fig. S2).

Sensitivity analyses were performed by using different models. We further adjusted separately for physical activity and occupation, and found that the estimated associations between blood plasma TMAO and related metabolites and the odds of asthenozoospermia were broadly consistent with the main analysis results (Supplementary Table S2).

## Discussion

The present case–control study indicated that increased blood plasma TMAO levels were positively associated with the odds of asthenozoospermia, while elevated levels of choline and L-carnitine were linked to the reduced odds. To the best of our knowledge, we are the first to establish a connection between TMAO and its metabolites and asthenozoospermia odds.

Dietary habits can affect TMAO concentrations (Koeth *et al.*, 2013; Janeiro *et al.*, 2018), which results in significant differences in its concentrations across countries and regions. In our study, the median blood plasma concentration of TMAO was 7.95 µmol/l, relatively higher when compared with results from a cross-sectional study (median 3.00 µmol/l) (Wu *et al.*, 2020) and a case–control study (median 1.47 µmol/l) (Shan *et al.*, 2017). This may be attributable to the fact that our study exclusively included male patients, whereas previous studies have demonstrated that blood plasma TMAO levels are elevated in males compared with females (Obeid *et al.*, 2017; Manor *et al.*, 2018; Barrea *et al.*, 2019). In contrast, the blood plasma TMAO level observed in our study was relatively lower than that found in a matched case–control study in Xinjiang, China (median: 8.59 µmol/l) (Ma *et al.*, 2024). Previous research has shown that prolonged consumption of red meat increases TMAO levels (Wang *et al.*, 2019), whereas the average red meat intake in Xinjiang is 133.0 g/day, substantially higher than the 81.0 g/day reported in our study (Zhang *et al.*, 2021; Fang *et al.*, 2023). Well-designed cohort studies have demonstrated that habitual intake of a relatively large amount of red meat is significantly associated with higher concentrations of TMAO (Buffa *et al.*, 2022; Li *et al.*, 2022). Long-term consumption of red meat can increase the levels of TMAO by three different mechanisms (Wang *et al.*, 2019): increasing nutrient density of dietary TMA precursors; enhancing microbial TMA/TMAO production from carnitine, but not choline; and reducing renal TMAO excretion. Stopping the consumption of red meat has been proven to reduce the blood plasma TMAO within 4 weeks (*Wang et al.*, 2019). Given that our study identified a multiplicative interaction between total red meat intake and all examined TMAO metabolites, it is crucial to focus on reducing red meat consumption, as it may act as a potential regulator of the relationship between TMAO metabolites and asthenozoospermia.

Our findings suggest that TMAO increases the odds of asthenozoospermia, while choline and L-carnitine reduce it. An analysis of 151 semen samples from normozoospermic men showed that TMAO may influence the methylation of the *H19-Igf2* gene to increase ROS levels, while high ROS levels reduce total sperm motility and sperm concentration (Darbandi *et al.*, 2019). An animal study using pigs has shown that TMAO is associated with sperm quality and function and could be used as a potential biomarker of sperm function (Mateo-Otero *et al.*, 2021). However, the above study was done using pig seminal plasma, while our results came from blood plasma. In addition, for the related metabolites of TMAO, choline is a key factor in regulating the sperm membrane structure and fluidity, and this nutrient plays a crucial role in the maturation and fertilizing ability of sperm (Lazaros *et al.*, 2012; Hanley, 2023). L-carnitine, as an antioxidant, has a protective effect on sperm motility in low temperature or low pressure and low oxygen environments (Chang *et al.*, 2023; Kaltsas, 2023; Nateghian *et al.*, 2023). Several animal studies have shown that betaine has a significant protective effect on sperm (Attia *et al.*, 2019; Billah *et al.*, 2022; Meng *et al.*, 2022; Mori *et al.*, 2022; Li *et al.*, 2024) and an improving effect on oligospermia (Lin *et al.*, 2024), but no correlation between betaine and asthenozoospermia was found in our study. Notably, different scholars hold opposite views on whether methionine damages or protects sperm. One study showed that methionine restriction mitigated the decline in sperm quality (Wu *et al.*, 2023), but another study verified that methionine restriction induced a decline in sperm viability and sperm transferred during mating (Wei *et al.*, 2024). Taken together, the relationship between these metabolites and asthenozoospermia remains inconsistent, and further studies are needed to evaluate this association.

TMAO and its metabolites may affect sperm function through a variety of mechanisms. TMAO can increase ROS production, which leads to lipid peroxidation of the sperm membrane, damages sperm DNA integrity, and induces OS (Darbandi *et al.*, 2019). Excessive OS can lead to changes in the testicular microenvironment and fragmentation of sperm DNA, which further contributes to asthenozoospermia (Arya *et al.*, 2022). Second, choline and other related metabolites of TMAO can reduce membrane stability and lead to abnormal membrane fluidity, thus affecting flagellar movement (Lazaros *et al.*, 2012; Hanley, 2023). Third, choline, betaine, and methionine can be used as methyl donors for DNA methylation, which plays an important role in male germ cell development and spermatogenesis (Lundy *et al.*, 2021). It has been confirmed that betaine can reverse the apoptosis of spermatogenic cells by increasing the methylation level of *Spata*, *Specc*, and *Spag* target genes through the PIWI/Pi-RNA pathway and up-regulation of methyltransferases, including DNA methyltransferases and histone methyltransferases (Lin *et al.*, 2024). Finally, evidence suggests that TMAO promotes vascular inflammation in mice by activating nuclear factor kappa B and subsequently increasing the expression of pro-inflammatory mediators (Tacconi *et al.*, 2023), and inflammatory processes can impair sperm maturation and migration, thereby impacting sperm function (Piomboni *et al.*, 2008; Liu *et al.*, 2021).

Our research has several strengths. First, to our knowledge, this is the first study to explore the relationship between blood plasma TMAO concentrations and its related metabolites and odds of asthenozoospermia. Second, while most previous studies have focused on measuring TMAO or just one of its metabolites, our study included TMAO along with several of its metabolites. By employing multiple indicators, we provided a more comprehensive perspective, thus enhancing the accuracy of the association between TMAO and its metabolites and asthenozoospermia odds. Third, we employed the LC-MS/MS method to quantify TMAO and its related metabolites in blood plasma samples. Due to its high accuracy and sensitivity, this technique provides reliable and valid data. Additionally, we matched each asthenozoospermia case with a healthy control by 1:1 to minimize the potential confounding effects.

There are still some limitations to our study. First, we cannot infer causality from this study due to the case–control study. Second, since the current study was conducted on a population of Chinese men, the extrapolated results may not accurately reflect other regions or populations. Third, a limitation of the current study lies in its relatively modest sample size. Although the statistical power of the primary analysis exceeded 90%, the sample size in the subgroup analysis may still have been insufficient. Fourth, our findings may have been influenced by other factors that can contribute to asthenozoospermia, including exposure to environmental pollutants (Szabo *et al.*, 2023), cellular and molecular factors (Shahrokhi *et al.*, 2020), as well as genetic causes (Heidary *et al.*, 2020), all of which were not assessed in this study. Lastly, blood plasma TMAO and its metabolites were measured at a single time point and may not accurately represent long-term concentrations, which may not fully capture the enduring effects on sperm quality. Nevertheless, large well-designed cohort studies are warranted in the future to explore whether there is a key association between TMAO and its related metabolites and risk of asthenozoospermia.

## Conclusion

In this case–control study, elevated blood plasma TMAO levels were associated with increased odds of asthenozoospermia, while higher concentrations of choline and L-carnitine decreased asthenozoospermia odds. These findings may serve as a foundation for future investigations aimed at exploring causality and developing novel metabolome-based diagnostics and therapeutics for men suffering from this complex and emotionally challenging condition.

## Supplementary Material

hoaf045_Supplementary_Data

## Data Availability

The data that support the findings of our study are available from the corresponding author upon reasonable request.
