# Supplementary material for: Blood plasma trimethylamine N-oxide and related metabolites and asthenozoospermia odds: a hospital-based matched case–control study in China
Source: Hum Reprod Open. 2025 Aug 18;2025(3):hoaf045. doi: 10.1093/hropen/hoaf045 (PMC12373642; doi:10.1093/hropen/hoaf045)
Supplement: hoaf045_Supplementary_Data [file hoaf045_supplementary_data.zip › Supplementary Figure S1-2_final.pdf]

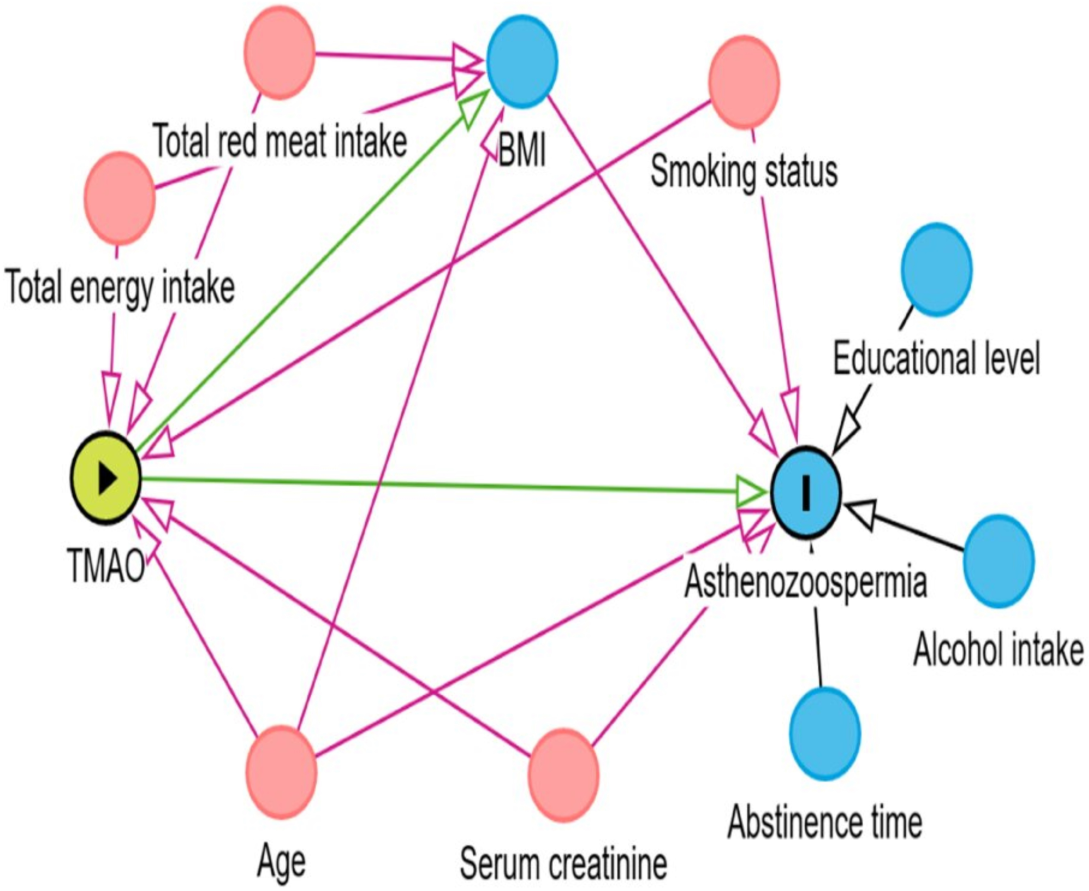

Supplementary Figure S1. Directed acyclic graph for the association between blood plasma trimethylamine N-oxide and related metabolites and asthenozoospermia.

Pink circles represent ancestors of the exposure and outcome (i.e., confounders), blue circles denote ancestors of the outcome (i.e., causal determinants of the outcome), and the green circle signifies the exposure factor. Green lines represent expected causal paths, and pink lines represent potential confounding paths, and black lines represent the association between confounders and the outcome; arrows at the end of each line represent the expected directionality of the association. The minimally sufficient adjustment set represents covariates such that the adjustment for this set of variables will minimize confounding bias when estimating the association between the exposure and the outcome. The minimally sufficient adjustment set was determined using the DAGitty website (https://www.dagitty.net/dags.html). The final minimally

sufficient adjustment set comprised educational level, total energy, and alcohol intake, serum creatinine, total red meat intake, and sexual abstinence time.

Abbreviation: TMAO, trimethylamine N-oxide.

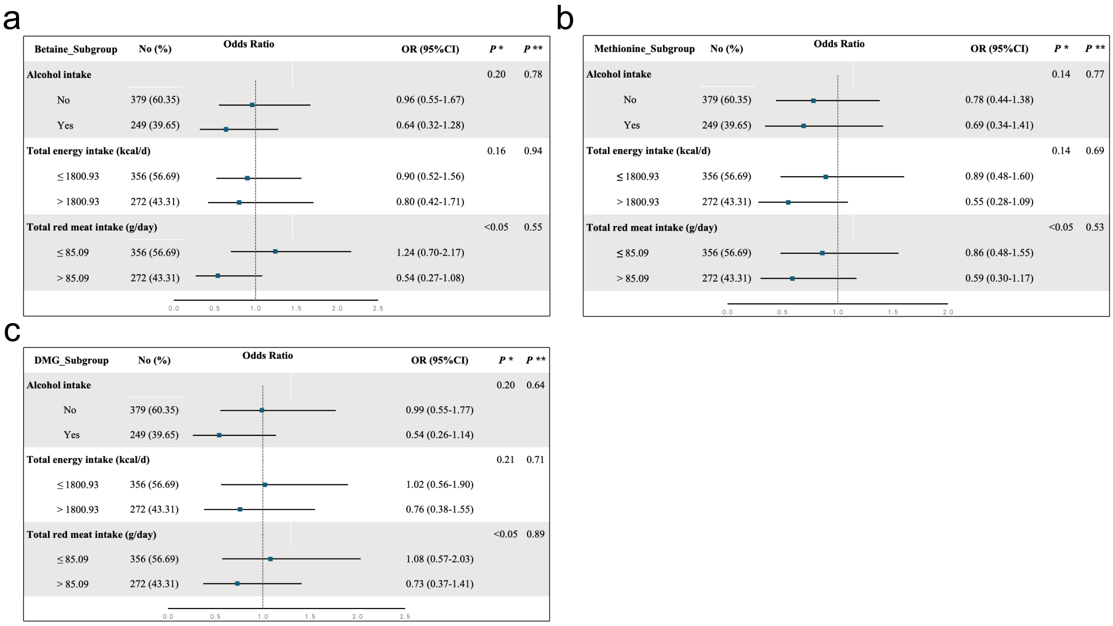

Supplementary Figure S2. Subgroup analyses of the associations between betaine, methionine, and DMG and the odds of asthenozoospermia.

(a) Subgroup analyses of the association between betaine and the odds of asthenozoospermia. (b) Subgroup analyses of the association between methionine and the odds of asthenozoospermia. (c) Subgroup analyses of the association between DMG and the odds of asthenozoospermia.

Abbreviations: CI, confidence interval; DMG, dimethylglycine; kcal/d, total energy intake of one day; OR, odds ratio.

* *P* for multiplicative interaction.

** *P* for additive interaction.

P-values <0.05 were considered statistically significant.
