# Supplementary material for: Blood plasma trimethylamine N-oxide and related metabolites and asthenozoospermia odds: a hospital-based matched case–control study in China
Source: Hum Reprod Open. 2025 Aug 18;2025(3):hoaf045. doi: 10.1093/hropen/hoaf045 (PMC12373642; doi:10.1093/hropen/hoaf045)
Supplement: hoaf045_Supplementary_Data [file hoaf045_supplementary_data.zip › Supplementary Tables_clean_final.docx]

**Supplementary Table S1. Subgroup analyses of the associations between TMAO and related metabolites and the odds of developing asthenozoospermia.**

|  |  | **TMAO** | **Choline** | **Betaine** | **L-carnitine** | **Methionine** | **DMG** | **Methyl donors** |
| --- | --- | --- | --- | --- | --- | --- | --- | --- |
| **Subgroup** | **Total** | **Quartile 4** | **Quartile 4** | **Quartile 4** | **Quartile 4** | **Quartile 4** | **Quartile 4** | **Quartile 4** |
| **Alcohol drinking** | | | | | | | | |
| No | 379 | 1.87 (1.05-3.31) | 0.50 (0.28-0.90) | 0.96 (0.55-1.67) | 0.64 (0.36-1.12) | 0.78 (0.44-1.38) | 0.99 (0.55-1.77) | 0.66 (0.37-1.17) |
| Yes | 249 | 1.56 (0.77-3.16) | 0.78 (0.39-1.57) | 0.64 (0.32-1.28) | 0.62 (0.30-1.28) | 0.69 (0.34-1.41) | 0.54 (0.26-1.14) | 0.59 (0.27-1.28) |
| * *P* |  | 0.30 | 0.13 | 0.20 | 0.11 | 0.14 | 0.20 | 0.09 |
| ** *P* |  | 0.35 | 0.62 | 0.78 | 0.90 | 0.77 | 0.64 | 0.71 |
| **Total energy intake (kcal/day)** | | | | | | | | |
| ≤ 1800.93 | 356 | 2.26 (1.24-4.15) | 0.54 (0.29-1.00) | 0.90 (0.52-1.56) | 0.77 (0.43-1.36) | 0.89 (0.48-1.60) | 1.02 (0.56-1.90) | 0.88 (0.48-1.61) |
| > 1800.93 | 272 | 1.34 (0.67-2.68) | 0.59 (0.29-1.20) | 0.80 (0.42-1.71) | 0.35 (0.17-0.72) | 0.55 (0.28-1.09) | 0.76 (0.38-1.55) | 0.51 (0.25-1.03) |
| * *P* |  | 0.81 | 0.18 | 0.16 | 0.13 | 0.14 | 0.21 | 0.13 |
| ** *P* |  | 0.56 | 0.85 | 0.94 | 0.36 | 0.69 | 0.71 | 0.40 |
| **Total red meat intake (g/day)** | | | | | | | | |
| ≤ 85.09 | 356 | 1.49 (0.84-2.65) | 0.57 (0.32-1.03) | 1.24 (0.70-2.17) | 0.59 (0.33-1.07) | 0.86 (0.48-1.55) | 1.08 (0.57-2.03) | 0.96 (0.53-1.75) |
| > 85.09 | 272 | 2.13 (1.04-4.36) | 0.58 (0.29-1.18) | 0.54 (0.27-1.08) | 0.49 (0.24-0.99) | 0.59 (0.30-1.17) | 0.73 (0.37-1.41) | 0.41 (0.20-0.83) |
| * *P* |  | 0.52 | < 0.05 | < 0.05 | < 0.05 | < 0.05 | < 0.05 | < 0.05 |
| ** *P* |  | 0.46 | 0.21 | 0.55 | 0.36 | 0.53 | 0.89 | 0.81 |

Quartile values for each model represent OR (95% CI). *P­-*values <0.05 were considered statistically significant.

Abbreviations: CI, confidence intervals; DMG, dimethylglycine; OR, odds ratios; TMAO, trimethylamine-N-oxide.

* *P* for multiplicative interaction.

** *P* for additive interaction.

**Supplementary Table S2. Sensitivity analyses between blood plasma TMAO and related metabolites and the odds of asthenozoospermia.**

|  | **Quartile 1** | **Quartile 2** | **Quartile 3** | **Quartile 4** | ***P* for trend** | **Per SD increment** |
| --- | --- | --- | --- | --- | --- | --- |
| **TMAO** |  |  |  |  |  |  |
| OR (95%CI) ^a^ | 1.00 (Ref) | 0.88 (0.55-1.42) | 1.17 (0.74-1.86) | 1.80 (1.16-2.80) | < 0.05 | 1.31 (1.12-1.54) |
| OR (95%CI) ^b^ | 1.00 (Ref) | 0.88 (0.54-1.42) | 1.17 (0.74-1.86) | 1.81 (1.16-2.81) | < 0.05 | 1.31 (1.12-1.55) |
| **Choline** |  |  |  |  |  |  |
| OR (95%CI) ^a^ | 1.00 (Ref) | 0.68 (0.44-1.06) | 0.81 (0.53-1.25) | 0.59 (0.37-0.93) | 0.06 | 0.82 (0.70-0.96) |
| OR (95%CI) ^b^ | 1.00 (Ref) | 0.68 (0.44-1.06) | 0.82 (0.53-1.26) | 0.58 (0.37-0.92) | 0.06 | 0.82 (0.69-0.96) |
| **Betaine** |  |  |  |  |  |  |
| OR (95%CI) ^a^ | 1.00 (Ref) | 0.68 (0.44-1.06) | 0.61 (0.39-0.95) | 0.84 (0.54-1.30) | 0.36 | 0.92 (0.79-1.08) |
| OR (95%CI) ^b^ | 1.00 (Ref) | 0.67 (0.43-1.05) | 0.60 (0.38-0.94) | 0.83 (0.54-1.28) | 0.33 | 0.92 (0.78-1.08) |
| **L-carnitine** |  |  |  |  |  |  |
| OR (95%CI) ^a^ | 1.00 (Ref) | 0.56 (0.36-0.87) | 0.55 (0.35-0.85) | 0.58 (0.37-0.90) | < 0.05 | 0.79 (0.67-0.93) |
| OR (95%CI) ^b^ | 1.00 (Ref) | 0.56 (0.36-0.87) | 0.55 (0.35-0.85) | 0.57 (0.37-0.90) | < 0.05 | 0.79 (0.67-0.93) |
| **Methionine** |  |  |  |  |  |  |
| OR (95%CI) ^a^ | 1.00 (Ref) | 0.91 (0.59-1.40) | 0.66 (0.42-1.03) | 0.77 (0.49-1.20) | 0.13 | 0.83 (0.70-0.97) |
| OR (95%CI) ^b^ | 1.00 (Ref) | 0.92 (0.59-1.41) | 0.66 (0.42-1.05) | 0.77 (0.49-1.20) | 0.13 | 0.83 (0.70-0.97) |
| **DMG** |  |  |  |  |  |  |
| OR (95%CI) ^a^ | 1.00 (Ref) | 0.94 (0.60-1.46) | 0.93 (0.60-1.45) | 0.84 (0.53-1.32) | 0.57 | 0.92 (0.78-1.08) |
| OR (95%CI) ^b^ | 1.00 (Ref) | 0.93 (0.60-1.46) | 0.93 (0.60-1.45) | 0.84 (0.53-1.31) | 0.58 | 0.92 (0.78-1.08) |
| **Total methyl donors** |  |  |  |  |  |  |
| OR (95%CI) ^a^ | 1.00 (Ref) | 0.92 (0.60-1.42) | 0.62 (0.39-0.97) | 0.71 (0.45-1.10) | < 0.05 | 0.82 (0.70-0.97) |
| OR (95%CI) ^b^ | 1.00 (Ref) | 0.91 (0.59-1.41) | 0.62 (0.39-0.97) | 0.70 (0.45-1.09) | < 0.05 | 0.82 (0.70-0.96) |

*P­-*values <0.05 were considered statistically significant.

Abbreviations: CI, confidential interval; DMG, dimethylglycine; OR, odds ratio; Ref, reference; SD, standard deviation; TMAO, trimethylamine-N-oxide.

^a^ Adjusted for educational level, total energy, alcohol intake, serum creatinine, total red meat intake, sexual abstinence time, and physical activity.

^b^ Adjusted for educational level, total energy, alcohol intake, serum creatinine, total red meat intake, sexual abstinence time, and occupation.
